# Extracts from the “News Letter”

**Published:** 1856-01

**Authors:** 


A late number of the *News Letter* gives a new method of using gold foil, by Prof. Arthur, which he considers of great

*I took models of Mr. P——'s upper and under jaws, and will send them, with the casts figured in my last communication, for the museum of the Baltimore College, when there is any chance of their going safely.
J. L. L.

practical utility. He has found that, re-annealing the gold foil after it is prepared in small blocks for filling, gives the gold greater adhesiveness, &c. It is known that foil, after laying for some time, and being handled, loses much of its adhesive property; and hence many in the profession have been in the habit of re-annealing the foil before using it. Prof. Arthur remarks, that

"In making some experiments for the purpose of testing the relative solidity, when condensed in a cavity, of gold foil and 'sponge gold,' I found the foil may be made to work in precisely the same way as the sponge gold, and as perfect adhesion obtained between the different portions of gold put into a cavity as with this material. Nothing more is necessary than to cut the sheets of the ordinary numbers of foil into two or three pieces, roll up very loosely, and hold it in the flame of a spirit lamp until it reaches a red heat. It will at once be found to have become so adhesive, that, with small and sharply serrated instruments, it may be made to adhere as readily as the best specimens of 'sponge gold.' It is necessary, however, when gold foil is used in this way, that it should be cut into small portions; for the very adhesiveness which it acquires when treated as I have proposed, prevents it from working well, if an attempt is made to use it in the ordinary manner.

"The instruments which I have found best adapted to the use of gold in this way, are small flattened pluggers, with two sharp points cut at the end, and made so hard that they will not turn. The gold may be picked up with such instruments, carried to the desired place, and condensed precisely as 'sponge gold.' It is well to go over each piece added, with a single sharp point.

"Although I have been making use of gold foil in this way for only about a month, I am exceedingly gratified at the results I have obtained, and am urged to call attention to it.

"It seems very strange that so desirable an improvement as this should have been in our hands for so long a time, and yet not discovered. I presume many operators have attempted to restore gold foil, which had become impaired, by re-heating it. I did so myself years ago, but found that the very adhesiveness it acquired by this process, interfered seriously with its use in the ordinary way.

"I have felt it necessary to make this long statement, in order to protect myself against the charge of vacillation, as I have at various times earnestly advocated several different methods of filling teeth. My strong desire has always been to improve, for my own benefit and that of others, the methods of performing the operations of our profession. I always have been, and always shall be, ready to take hold of any new thing which promises advantages, and to test it as thoroughly as I am capable of doing. I have not changed my ground in relation to any of the forms of gold I have advised to be used. Each has certainly the advantages which I attributed to them. I do not now take the ground that I have been mistaken in my expressed views of 'sponge gold;" I still regard it as perfectly reliable, when properly used. But for ordinary use, I am convinced that gold, in the form I am now recommending it, is a decided improvement upon any method of using gold hitherto known. Whether it will be regarded as superior to 'sponge gold,' in the hands of those who have successfully used this material, is a question which time and trial can alone decide.

"I have no idea that gold will be used generally in the manner I now recommend. It is exceedingly difficult to induce men to change a course which they have successfully pursued for years, and the difficulties of which they have learned to encounter and overcome, for any new thing; and, even if they are disposed to make trial of a new process, it is often so imperfect a trial, that it is unsatisfactory. With regard to the matter in hand, simple as it is, I confidently say to every operator in the profession that, if fairly tried, it will afford advantages in the use of gold foil of which few have dreamed.

"I may say that the foil of different manufacturers presents a remarkable difference in regard to this quality of adhesiveness. It is certainly advisable to try several kinds, if that in hand does not work well. The profession may rely upon the full truth of the statements I have made. I have never, in the course of practice of fifteen years, made use of any material for filling teeth which has so fully satisfied me as foil used in the manner I have described."

The October number of the *News Letter*, for 1855, contains an article from Dr. Chase, on the Springing of Plates, which he "feels" is "absolutely certain," and that "the result may be relied upon."

We really cannot see the rationale of the operation, yet, coming from so good a source, we feel that it should be tried. There are certain known effects of heat which we have supposed all the investments which can be applied, could not prevent; hence our object is to apply that heat so as not to exert an undue influence on the plate. We cannot see how a gold plate can be heated to nearly fusion point, and yet retain its exact position, or settle back to it when cooling. If such be the fact, there must be an adaptation of solder and plate, so as not to require that amount of heat, or the plate will be sprung. But we give the Dr.'s method:

"My method is this:—After the teeth are fitted to the plate, I take a strip of copper, one inch and a quarter in width, and about No. 23 of the gauge in thickness, and wrap it round the case, allowing the copper to come within one-eighth of an inch of the teeth; I then cut it off so that the two ends may be fastened; this is done by punching a hole in each end of the copper and then putting through a piece of iron, copper or platina wire, and twisting the ends. Before fastening the ends of the copper plate, however, I cut into it on one side, making a sort of fringe, leaving the strips about one-eighth or three-sixteenths of an inch in width. Either the whole or a part of every third strip is cut out. Now fasten the ends of the copper plate together, making the shape to correspond with that of the case; then turn the 'fringe,' with a pair of pliers or otherwise, inwards, at right angles, thereby making a sort of cup just deep enough for your case to set in, without having the cutting or grinding surfaces of the teeth above the upper border of the copper plate.

"Put the 'case' into the 'cup.' Mix plaster and sand very thin, using very little plaster, and pour between the teeth and the sides of the cup. If the teeth and the inside of the cup are oiled, the plaster will run more freely. Put not a particle of plaster either on the lingual or palatine surface of the plate. After a few moments the wax may be removed, and the teeth backed, if it has not already been done. I prefer to back them after they are put into the 'cup.' Some one of the teeth may always be easier removed from the plaster than the rest, and after that is out, there is no difficulty in taking them all out. I never put any plaster on the cutting or grinding surfaces of the teeth. The depth of the cup may be enlarged or contracted by altering the position of the 'fringe.'"

